# Anticancer effect of hUC-MSC-derived exosome-mediated delivery of PMO-miR-146b-5p in colorectal cancer

**DOI:** 10.1007/s13346-023-01469-7

**Published:** 2023-11-17

**Authors:** Siming Yu, Ran Liao, Lu Bai, Madi Guo, Yu Zhang, Yumin Zhang, Qi Yang, Yushuai Song, Zhiwei Li, Qingwei Meng, Shubin Wang, Xiaoyi Huang

**Affiliations:** 1 Department of Pharmacy, Guangdong Province, Peking University Shenzhen Hospital, Shenzhen, 518036 People’s Republic of China; 2 Department of Pharmacy, PKU-Shenzhen Clinical Institute of Shantou University Medical College, Shenzhen, People’s Republic of China; 3 Biotherapy Center, Harbin Medical University Cancer Hospital, Heilongjiang Province, Harbin, 150081 People’s Republic of China; 4 Department of Laboratory, Lianyungang Maternal and Child Health Care Hospital, Jiangsu Province, Lianyungang, 222000 People’s Republic of China; 5 Department of Gastrointestinal Medical Oncology, Harbin Medical University Cancer Hospital, Heilongjiang Province, Harbin, 150081 People’s Republic of China; 6 Department of Medical Oncology, Harbin Medical University Cancer Hospital, Heilongjiang Province, Harbin City, 150081 People’s Republic of China; 7 Department of Oncology, Guangdong Province, Shenzhen Key Laboratory of Gastrointestinal Cancer Translational Research, Cancer Institute, Peking University Shenzhen Hospital, Shenzhen-Peking University-Hong Kong University of Science and Technology Medical Center, Shenzhen, 518036 People’s Republic of China; 8 NHC Key Laboratory of Cell Transplantation, Harbin Medical University, Heilongjiang Province, Harbin, 150081 People’s Republic of China

**Keywords:** Colorectal cancer, Drug delivery, Epithelial-mesenchymal transition, Exosomes, hUC-MSCs

## Abstract

Antisense oligonucleotide (ASO) is a novel therapeutic platform for targeted cancer therapy. Previously, we have demonstrated that miR-146b-5p plays an important role in colorectal cancer progression. However, a safe and effective strategy for delivery of an ASO to its targeted RNA remains as a major hurdle in translational advances. Human umbilical cord mesenchymal cell (hUC-MSC)–derived exosomes were used as vehicles to deliver an anti-miR-146b-5p ASO (PMO-146b). PMO-146b was assembled onto the surface of exosomes (e) through covalent conjugation to an anchor peptide CP05 (P) that recognized an exosomal surface marker, CD63, forming a complex named ePPMO-146b. After ePPMO-146b treatment, cell proliferation, uptake ability, and migration assays were performed, and epithelial-mesenchymal transition progression was evaluated in vitro. A mouse xenograft model was used to determine the antitumor effect and distribution of ePPMO-146b in vivo. ePPMO-146b was taken up by SW620 cells and effectively inhibited cell proliferation and migration*.* The conjugate also exerted antitumor efficacy in a xenograft mouse model of colon cancer by systematic administration, where PPMO-146b was enriched in tumor tissue. Our study highlights the potential of hUC-MSC-derived exosomes anchored with PPMO-146b as a novel safe and effective approach for PMO backboned ASO delivery.

**Graphical Abstract:**

Schematic illustration of the preparation of an exosomal anchor peptide (CP05)-PMO that conjugately binds to exosomes from hUC-MSCs (ePPMO-146b) and the antitumor effect of ePPMO-146b in CRC, which occurs through the inhibition of Smad signaling and epithelial–mesenchymal transition.

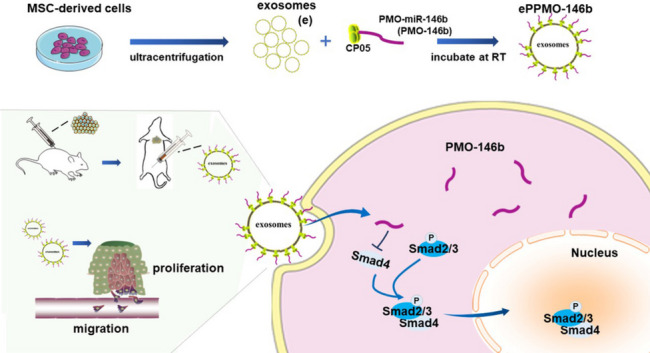

**Supplementary Information:**

The online version contains supplementary material available at 10.1007/s13346-023-01469-7.

## Introduction

Over the past decades, the focus of the pharmaceutical and biotechnology industries on anticancer agents has transitioned from traditional cytotoxic drugs to precisely targeted therapies. Advances in nanotechnology have shed light on engineered agents, which offer the potential for crossing biological barriers and performing transmembrane deliveries. Exosomes are a class of natural biological nanoparticles; are the smallest extracellular vesicles [1], with sizes falling between 50 and 150 nm; and are secreted from different types of cells through the endolysosomal pathway [2, 3]. Nanosized vesicles have the capacity to carry a wide variety of cargoes such as nanodrugs, functional proteins, and antisense oligonucleotides (ASOs), like miRNAs, anti-miRNAs, and small interfering RNAs (siRNAs) [4]. Due to their low immunogenicity, low toxicity, good biocompatibility, ability to be biodistributed to specific cells and tissue, and ability to transfer their contents into recipient cells via endocytosis, exosomes are leveraged on these characteristics to be ideal delivery strategies of therapeutic molecules as cell-free therapeutics [5, 6].

Mesenchymal stem cells (MSCs) are easily accessible and can be derived from different sources such as bone marrow, placenta, adipose tissue, and umbilical cord; therefore, MSCs are believed to be the most prolific producers of exosomes [7, 8]. MSC-derived exosomes as vehicles that can carry therapeutic molecules such as miRNA and siRNA have been widely explored. Lou et al. observed that miR-199a-modified exosomes from adipose tissue-derived MSCs effectively sensitized hepatocellular carcinoma (HCC) to chemotherapies by targeting the mTOR pathway [9]. Another group reported that siGRP78-modified bone marrow MSC (BM-MSC)-derived exosomes could reverse the drug resistance of HCC cells to sorafenib [10]. The first engineered MSC-derived exosomal therapy that carried KRAS G12D siRNA has entered clinical trials to treat pancreatic cancer (NCT03608631) [11]. In addition, this engineered exosomal platform that carried STAT3 siRNA or an ASO showed potential to ameliorate liver fibrosis [12]. Despite the therapeutic value of modified MSC-derived exosomes, their translation to clinical practice is still hindered by the search for a feasible approach that enhances the in vivo delivery efficacy and supports convenient and sustainable production.

Colorectal cancer (CRC) is the third leading cause of cancer mortality worldwide [13, 14]. Even after chemotherapy, the prognosis of metastatic CRC is still poor, and the 5-year survival rate is less than 20% because of the toxicity and poor tolerance of patients to chemotherapy [14]. Hundreds of studies have indicated that microRNAs (miRNAs) are key regulators in CRC cell invasion and metastasis by facilitating epithelial-to-mesenchymal transition [15–17]. Epithelial-mesenchymal transition is essential for promoting tumor invasion and metastasis in CRC [18]. We previously reported that miR-146b-5p (miR-146b) significantly promoted epithelial–mesenchymal transition phenotypes through Smad4 in CRC cells [19]. An in vivo study has demonstrated that miR-146b may play a tumor-promoting role in establishment of CRC [20, 21]. To therapeutically modulate miR-146b expression, we employed a synthetic anti-miR-146b ASO (PMO-146b) that is complementary to the mature miR-146b. Synthetic anti-miRNA oligonucleotides (PMOs) are believed as a new class of therapeutic agents specifically inhibiting individual miRNAs. However, the major challenge in applying PMO for clinical use is its insufficient cellular uptake [22, 23].

Herein, we evaluated whether the hUC-MSC-derived exosome carrying PMO-146b could inhibit CRC progression. PMO-146b is a phosphorodiamidate morpholino oligomer (PMO) [1, 24]. Quality-controllable exosomes that were sustainably generated by hUC-MSCs were verified by performing nanoflow cytometry (nanoFCM). We then loaded the PMO-146b conjugated to the CP05 peptide (CRHSQMTVTSRL) on the surface of exosome. CP05 peptide has been shown to bind to tetraspanin CD63 enriched on the exosomal membrane [25–27]. Systemic administration of exosomes loaded the CP05 and PMO-146b conjugate (ePPMO-146b) was evaluated for safety and efficacy both in vitro and in vivo and demonstrated a therapeutic effect in a CRC mouse model. The present study indicates that MSC-derived exosome anchored with an ASO may serve as a promising strategy for CRC therapy.

## Material and methods

### MSCs and tumor cell lines

Human umbilical cord MSCs (hUC-MSCs) were obtained from the Biotherapy Center of Harbin Medical University Cancer Hospital and prepared following a well-established protocol [28]. The human umbilical cords were donated by women who had underwent eutocia. Informed consent was obtained from the subjects’ families and the study was approved by the Harbin Medical University Cancer Hospital Ethics Review Board. Purity was confirmed by flow cytometry (CD73 + , CD90 + , CD105 + , CD146 + , CD31-, CD34-, CD45-, and HLA-DR-). These MSCs can differentiate into osteoblasts, chondroblasts, and adipocytes in vitro. MesenCult™ MSC Basal Medium (Stem Cell, USA) was used to culture MSCs without serum in a GMP-grade condition. The 4^th^ passage was used for the following experiments. The differentiation of MSCs to adipocytes, osteocytes, and chondrocytes was tested by using StemPro^®^ and an adipogenesis kit (cat No. A10070-01, Gibco, USA), osteogenesis kit (cat No. A10072-01, Gibco, USA), and chondrogenesis differentiation kit (cat No. A10071-01, Gibco, USA). Afterward, staining with Oil Red O, Alizarin Red S, and Alcian Blue was also performed to detect adipocytes, osteocytes, and chondrocytes, respectively.

The normal colonic mucosa cell line NCM460 was purchased from the Shanghai Cell Bank. The human HCT116 colorectal carcinoma (ATCC CCL-247™), SW480 adenocarcinoma carcinoma (ATCC CCL-228™), Caco2 adenocarcinoma (ATCC HTB-37™), Lovo colorectal adenocarcinoma (ATCC CCL‐229™), HT29 adenocarcinoma (ATCC HTB-38™), and SW620 colorectal adenocarcinoma (American Type Culture Collection (ATCC)^®^ CCL-227™) cell lines were purchased from the ATCC. With the exception of Caco2, the other cell lines were grown in DMEM (Gibco, USA) supplemented with 10% fetal bovine serum (FBS) and 1% antibiotic–antimycotic solution in a 5% CO_2_ incubator. Caco2 cells were cultured in DMEM supplemented with 20% FBS.

### Isolation and purification of research-grade exosomes

hUC-MSC-derived exosomes were purified by ultracentrifugation. Five hundred-milliliter supernatants were collected from MSCs cultured as monolayers in serum-free medium and were subsequently subjected to centrifugation at 3000 × g for 30 min. The supernatants were then filtered using 0.2-μm filters, and the pellet was recovered and subsequently ultracentrifuged (Beckman, USA) at 100,000 × g using a SW32 Ti rotor for 3 h. The supernatants were aspirated and the resulting pellet was suspended again in PBS and again centrifuged at 100,000 × g for 1 h. The supernatants were aspirated and the pellet was recovered in 2 mL PBS and stored at – 80 °C until use.

### Transmission electron microscopy (TEM)

The exosome suspension was diluted with PBS at a 1:1 ratio, and 10 μL of this solution was dropped onto formvar-carbon-coated grids and blotted with filter papers after sedation for 1 min. Then, 10 μL of 3% phosphotungstic acid was dropped onto the exosome area for 1 min. After the excess staining buffer was removed with filter papers, the grid was left to air-dry for 5 min. Exosome morphologies were visualized using a high-resolution transmission electron microscope (Hitachi HT7700, Japan) at 80 kV.

### Nanoflow cytometry (nanoFCM) for exosome size and concentration analysis

Flow NanoAnalyzer model type N30 (NanoFCM Inc., China) was used to determine the exosome size distribution and granular concentration according to the manufacturer’s instructions. Briefly, the isolated exosomes were diluted with PBS at a 1:100 ratio. The Silica Nanospheres Cocktail (S16M-Exo, NanoFCM Inc., China) was employed to construct a calibration curve regarding particle size and side scattering intensity. Using this calibration curve, the side scattering intensity of every exosome was converted into the corresponding vesicle size.

### Flow cytometry analysis of exosome-bound beads

Exosomes (1.32 × 10^10^ particles) from MSCs were isolated as described above and resuspended in 200 μL PBS. Aldehyde/sulfate latex beads (10 μL, A37304, Life Technologies, USA) were added to the solution and mixed using a benchtop rotator for 15 min at room temperature. PBS (600 μL) was then added to the solution and mixed overnight at 4 °C. The mixture was then spun down at 8000 × g for 1 min. The precipitate was then resuspended in 100 μL of 10% BSA in PBS and mixed for 45 min at room temperature. The mixture was spun down at 8000 × g for 1 min, and the supernatant was aspirated. Exosome-bound beads (the pellet) were then resuspended in 20 μL PBS and immunolabeled for CD63, CD73, CD90, or an isotype control. The beads were incubated with 1 μL anti-CD63 antibody (eBioscience, USA) or 1 μL anti-CD73 (Material Number 550257, BD Biosciences, USA) or 1 μL anti-CD90 antibody (Material Number 555596, BD Biosciences, USA) or 1 μL Mouse IgG1, κ isotype control antibody (Material Number 555749, BD Biosciences, USA) in a final volume of 20 μL and mixed at room temperature for 30 min in the dark. The mixture was then centrifuged at 8000 × g for 1 min, the supernatant was aspirated, and the pellet was resuspended in 200 μL PBS with 2% BSA. The expression of exosomal markers (CD63) and mesenchymal markers (CD73 and CD90) was analyzed using flow cytometry (BD FACSAria II analyzer, USA). Data were analyzed using FlowJo software (TreeStar Inc., USA). The flow cytometry experiment was repeated two times independently using the same exosome preparation.

### Binding identification of exosome and PMO

To measure the binding affinity of candidate peptides to exosomes, 1.32 × 10^10^ exosomes were preincubated with biotin-labeled CP05-anti-miR-146b-5p ASO (PPMO-146b) or biotin-labeled anti-miR-146b-5p ASO (PMO-146b; as a control for CP05) overnight at 4 °C (PPMO-146b or PMO-146b solution (10 μg/μL) resolved in PBS at 1 mM), followed by washing with PBS for five times in 1.5-mL ultracentrifuge tubes and filtration with Amicon Ultra-0.5 Centrifuge Filter (Millipore, USA) to remove unbound peptides (14,000 × g, 10 min). Subsequently, the exosome-PMO complexes, ePPMO-146b, and ePMO-146b were incubated with 4-μm aldehyde/sulfate latex beads for 15 min at room temperature under rotation and washed with PBS for three times (8000 × g, 1 min each time). The segregated complexes were then incubated with 3% BSA-DPBS for 30 min on a rotator. After washing with PBS for 3 times (8000 × g, 1 min), streptavidin-PE (1:500, diluted in 3% BSA blocking buffer, Catalog Number 12–4317, eBioscience, USA) was added to the complexes and incubated for 30 min at room temperature in the dark. After washing 3 times with PBS, the recovered beads were observed with a conventional fluorescence microscopy (Zeiss, Germany) or subjected to flow cytometry (FACSCalibur, BD, USA). Uncoated beads were used as negative controls for gating. We used the separation index (SI) metric that was defined by Theodoraki et. al, which takes into account both the difference in mean fluorescent intensity (MFI) between PPMO-146b and PMO-146b SI = $\frac{\left({\mathrm{MFI}}_{\mathrm{PPMO}-146\mathrm{b}}-{\mathrm{MFI}}_{\mathrm{PMO}}-146\mathrm{b }\right)}{\sqrt{\frac{{{\mathrm{SD}}_{\mathrm{PPMO}-146\mathrm{b}}}^{2}+{{\mathrm{SD}}_{\mathrm{PMO}-146\mathrm{b}}}^{2}}{2}}}$ capture beads and the average of their distributions [29]. PMO (Gene Tools LLC, USA) sequences are listed in Table 1. The peptide CRHSQMTVTSRL (CP05) was conjugated with PMO-146b (PPMO-146b) as previously described [25, 30].

**Table 1:** Oligonucleotide and primer sequences

Name	Sequence (5′-3′)
Negative control	CCTCTTACCTCAGTTACAATTTATA
has-anti-miR-146b-5p ASO	CACAGCCTATGGAATTCAGTTCTCA
miR-146b RT	GTCGTATCCAGTGCGTGTCGTGGAGTCGGCAATTGCACTGGATACGACAGCCTATG
miR-146b F	GGGCGGTGAGAACTGAATT
miR-146b R	CAGTGCGTGTCGTGGAGT
U6 F	CTCGCTTCGGCAGCACA
U6 R	AACGCTTCACGAATTTGCGT

All primers correspond to *Homo sapiens*

### Uptake of PKH67-labeled ePPMO-146b by SW620 cells

To determine whether SW620 CRC cells could take up targeted exosomes from hUC-MSCs, the PKH67 Green Fluorescent Cell Linker Kit (ThermoFisher, USA) was used to label exosomes, according to the manufacturer’s protocol. Briefly, MSC-derived exosomes, ePMO-146b-biotin, and ePPMO-146b-biotin were diluted and resuspended in sterile PBS to a final concentration of 5 × 10^7^ particles. Diluent C (500 μL; 2 × PKH67 solution) was added to 2-µL PKH67 dye (PKH67GL, Sigma, USA), and 500 μL exosomes were mixed with 500-μL PKH67 solution to allow internalization. Subsequently, incubate the exosomes/dye mixture for 1–5 min with periodic mixing, and then, the staining was stopped by adding an equal volume of serum or 1% BSA-PBS. Then, the unbound PKH67 was removed by using Amicon Ultra-0.5 Centrifuge Filter (Millipore, USA) at 14,000 × g for 10 min.

SW620 cells were seeded in 12-well plates at a density of 1 × 10^5^ cells/well and were incubated in a complete medium for 12 h. Subsequently, the plates were rinsed twice with PBS and fixed with 4% paraformaldehyde solution at 4 ℃. The plates were then rinsed again three times using PBS. After rewashing, 10 µM unlabeled avidin (S888, Invitrogen, USA) was added to link biotinylated PMO on the membrane surface for 30 min at 4 ℃.

Next, the cells were permeabilized with 0.1% Triton X-100 for 5 min. PBS with 3% BSA was used to incubate with the cells for 2 h to block nonspecific binding. Then, streptavidin R PE (SNN1007, Invitrogen, USA) was utilized for the detection of PMO-labeled biotinylated anti-miR-146b-5p ASO in the presence or absence of CP05 peptides at room temperature for 1 h. Cells incubated with native exosomes and PBS with the same volume as the suspension of PMO-146b-loaded exosomes served as controls. Finally, the cells were washed three times with precooled PBS and were mounted with DAPI-containing mounting media (H-1800, Vector Labs, USA). Images were captured under a fluorescence microscope (Zeiss, Germany).

### Quantitative real-time PCR

Total RNA was extracted from seven cell lines with TRIzol reagent (Invitrogen, USA). The cDNAs were produced from the RNA samples with PrimeScript™ 1st Strand cDNA Synthesis Kit (No. 6110A, TaKaRa, Japan). Human U6 snRNA was used as an endogenous control for data normalization. Real-time PCR was performed on the LightCycler^®^96 system (Roche, USA) using TB Green™ Premix Ex Taq™ (No. RR820L, Takara, Japan). Relative miRNA expression was determined using the Ct method. All experiments were performed at least three times. Oligonucleotides were synthesized by Integrated DNA Technologies (Sangon Biotech, China), and the primer sequences were designed as previously described [19]. The primer sequences are listed in Table 1.

### Cell viability analysis with a CCK8 assay

SW620, Caco2, and Lovo cells were seeded in 96-well plates at a density of 5–8 × 10^4^ cells/well. Once the cells reached an approximately about 80% confluency, they were starved overnight and cocultured with different concentrations of exosomes (2.6 × 10^9^, 2.6 × 10^10^, and 2.6 × 10^11^ particles/mL) or an equal volume of PBS. In another experiment, the cells were exposed to exosomes, ePNC at an exosomal concentration of 2.6 × 10^10^ particles/mL (scramble PMO fragment, negative control for PMO-146b); ePPMO-146b at an exosomal concentration of 2.6 × 10^10^, 1.3 × 10^10^, and 6.5 × 10^9^ particles/mL; or 5-FU at a concentration of 3 mM. Cell growth was analyzed 48 h after treatment. The data shown are representative of at least three independent experiments.

### Wound healing assay

SW620 and Caco2 cells were seeded onto a six‐well plate at a density of 5 × 10^5^ cells/mL until they reached full confluency. The cells were cultured for 24 h in the presence of exosomes, ePNC (exosome concentration, 2.6 × 10^10^ particles/mL), or ePPMO-146b (exosome concentration, 2.6 × 10^10^, 1.3 × 10^10^, and 6.5 × 10^9^ particles/mL). Then, each well was scratched by using a 200-μL pipette tube to create 2 linear regions that were devoid of cells, and medium without FBS was added. PBS was used as an additional negative control. Photographs were captured by a digital single lens reflex camera (Canon, Japan) at 0 and 24 h, respectively. The migration area of the cells was measured by ImageJ software.

### Transwell migration assay

SW620 and Caco2 cells (4–5 × 10^4^ cells/well) were seeded on upper chambers (6.5 mm Transwell^®^ with 8.0-μm pore polycarbonate membrane insert; Corning, USA) in serum-free medium with 0.1% BSA. A total of 600 μL medium containing 10% FBS was added to the lower chambers. The cells were cultured in the presence of 2.6 × 10^10^, 1.3 × 10^10^, and 6.5 × 10^9^ particles/mL exosomes. After incubation for 24 h, nonmigrating and noninvading cells were gently removed with a cotton swab. The cells were then fixed with methanol for 30 min and stained with 0.1% crystal violet for another 20 min. The area of dyed pores was then calculated under a microscope at a magnification of × 200. Three views were selected randomly for photography and analysis. Each measurement was repeated three times.

### Western blot analysis

Total proteins from cells or exosomes were extracted at 4 °C by using RIPA Lysis Buffer (Beyotime, China) with a protease inhibitor (Roche, USA) and were then vortexed every 5–10 min for 30 min. Subsequently, the lysates were spun down at 14,000 × g for 20 min to remove any debris and the supernatant was collected. Exosomes (20 μg) and protein samples (70 μg per lane) were loaded onto 10% SDS-PAGE gels and transferred onto PVDF membranes (Millipore, USA) for 90 min. For immunodetection, the membranes were incubated with the following primary antibodies at 4 °C overnight: CD63 (ab134045), CD81 (ab109201), TSG101 (ab125011), Syntaxin6 (10,841–1-AP), Erp72 (14,712–1-AP), Smad4 (10,231–1-AP), E-cadherin (ab76055), vimentin (ab92547), and N-cadherin (ab76011). All primary antibodies were purchased from Abcam (Cambridge, UK) or Proteintech (Wuhan, China). The next morning, the membranes were washed with TBS-T three times for 10 min each time. After incubation with secondary antibody at room temperature for 1 h, the membranes were washed with TBS-T and then incubated with luminol substrate solution (Transgene, China) for 1 min. Images were collected with a chemiluminescence imaging system (ProteinSimple, USA). All experiments were performed at least three times.

### In vivo antitumor efficacy of ePPMO-146b in tumor-xenografted nude mice

Adult female athymic BALB/c nude mice (15–20 g) that were 8 weeks old were purchased from Beijing Vital River Laboratory Animal Technology Co., Ltd. (Certificate No. 110011211106810132 SCXK (Jing) 2021–0006). The animals were housed in a controlled environment at 23 ± 2 °C under a 12-h dark/light cycle with free access to irradiated food and sterile water. To ensure the number of tumor-bearing mice, 10 female BALB/c nude mice were subcutaneously injected with Lovo cells in 200 μL of PBS (2 × 10^6^ cells). One week later, the tumor-bearing mice were approximately 90 mm^3^ in size. The tumor-bearing mice were randomly divided into an ePNC group and ePPMO-146b group (*n* = 5) on the basis of their tumor volume. ePNC solution and ePPMO-146b solution (5.0 × 10^10^ particles/kg in 10 μL) were injected into the tumor-bearing mice via a peritoneal injection. The mice were treated twice a week for 24 days and tumor size was measured once or twice a week for 24 days with a vernier caliper. Tumor volumes were calculated according to the following formula: volume (mm^3^) = length × (width)^2^/2. The mice were killed at 25 days post-injection of ePNC and ePPMO-146b. The inhibitory rate of tumor volume was calculated as IR (%) = (*V*_t_ − *V*_0_)/*V*_0_ × 100%, where *V*_0_ is the tumor volume of the ePNC group mice and *V*_t_ is the tumor volume of the ePPMO-146b group. The inhibitory rate of tumor weight was calculated as IR (%) = (*W*_t_ − *W*_0_)/*W*_t_ × 100%, where *W*_t_ and *W*_0_ represent the tumor weights of the ePNC and ePPMO-146b-treated groups, respectively.

### Hematoxylin and eosin and immunohistochemistry analysis

The extracted tumor, liver, spleen, kidney, lung, and heart were sequentially fixed with 4% paraformaldehyde and embedded with paraffin. The tumor paraffin sections were used for immunohistochemistry (IHC) assay and the other tissues were incubated at 45 ℃ for 2 h for hematoxylin and eosin (H&E) staining.

For IHC assay, heat-induced epitope retrieval was performed by use of Citrate Antigen Retrieval Solution (Solarbio, China) for 40 min for vimentin or by EDTA Antigen Retrieval Solution (Abcam, China) for E-cadherin. The tissue slides were incubated with the primary antibodies for E-cadherin (1:500; #14472S; Cell Signaling Technology, Danvers, MA, USA) and vimentin (1:250; HPA001762; Sigma-Aldrich) at 4 °C overnight, followed by incubation with the secondary antibody EnVision™ + /HRP mouse (rabbit) polymer (Dako, Denmark) at room temperature for 30 min. Secondary antibody detection was performed by using the SIGMAFAST™ 3,30-diaminobenzidine tablets (DAB Peroxidase Substrate Tablet Set) (D4168; Sigma-Aldrich). Slides were counterstained with hematoxylin for 2 min following color separation by 1% acetic acid for 30 s.

For H&E staining, the tissue sections were then treated with gradient concentrations of xylene and ethanol. After washing with water, the tissue sections were stained with hematoxylin for 10 min, washed with tap water, and then treated with 1% hydrochloric acid ethanol. Following dehydration of ethanol, the tissue sections were stained with eosin solution, dehydrated, and sealed with mounting medium. The slides were observed under a light microscope (Leica DM750, Germany).

### Biodistribution of systemical ePPMO-146b

DiR-labeled exosomes were prepared according to the literature [31]. Purified exosomes (5.0 × 10^9^ particles/mL) were incubated with 1 μM fluorescent lipophilic tracer DiR (D12731, Invitrogen, USA) for 5 min at 37 ℃ prior to 4 ℃ for another 15 min. Unbound dye was removed by centrifugation at 100,000 × g overnight by means of an SW41 Ti rotor (Backman), and then, the pellet was washed twice with precooled PBS. The obtained DiR-Exos were resuspended in precooled PBS, and the particle concentration was measured by nanoFCM. DiR-Exos were diluted in PBS to achieve a particle concentration of 8.0 × 10^9^/mL.

Female healthy male C57BL/6N mice (Animal Center of the 2nd Affiliated Hospital of Harbin Medical University) or C57BL/6 nude mice aged 8 weeks were used. Freshly purified DiR-labeled exosomes and naked exosomes were injected intraperitoneally (i.p.) in the C57BL/6N mice. The biodistribution of DiR-labeled exosomes was examined using the same dosage mentioned above, and the particle count was measured with nanoFCM and the samples were diluted to 200 μL. To analyze DiR-exosome distribution, IVIS Spectrum (Perkin Elmer) was performed. Live mice (isoflurane sedated) were imaged after 3 h post-treatment. For ex vivo imaging, the mice were sacrificed after 6 h post-injection and the liver, heart, lung, kidney, spleen, intestine, and reproductive organs were collected. The live mice or the harvested organs were imaged for 1–2 s (at an excitation wavelength of 640 nm and an emission wavelength of 710 nm).

DiR-ePPMO-146b was intraperitoneally injected in the tumor-bearing C57BL/6 nude mice. At 6 h post-injection (5.0 × 10^10^ particles/kg), organs were harvested and prepared as described above.

### Statistical analysis

Statistical comparisons among multiple groups were determined by one-way analysis of variance (ANOVA) with Dunnett’s post hoc test. A probability value of 0.05 was considered to be significant. The results are expressed as the mean ± SEM.

## Results

### Characterization of MSCs and MSC-derived exosomes

**Fig. 1:**
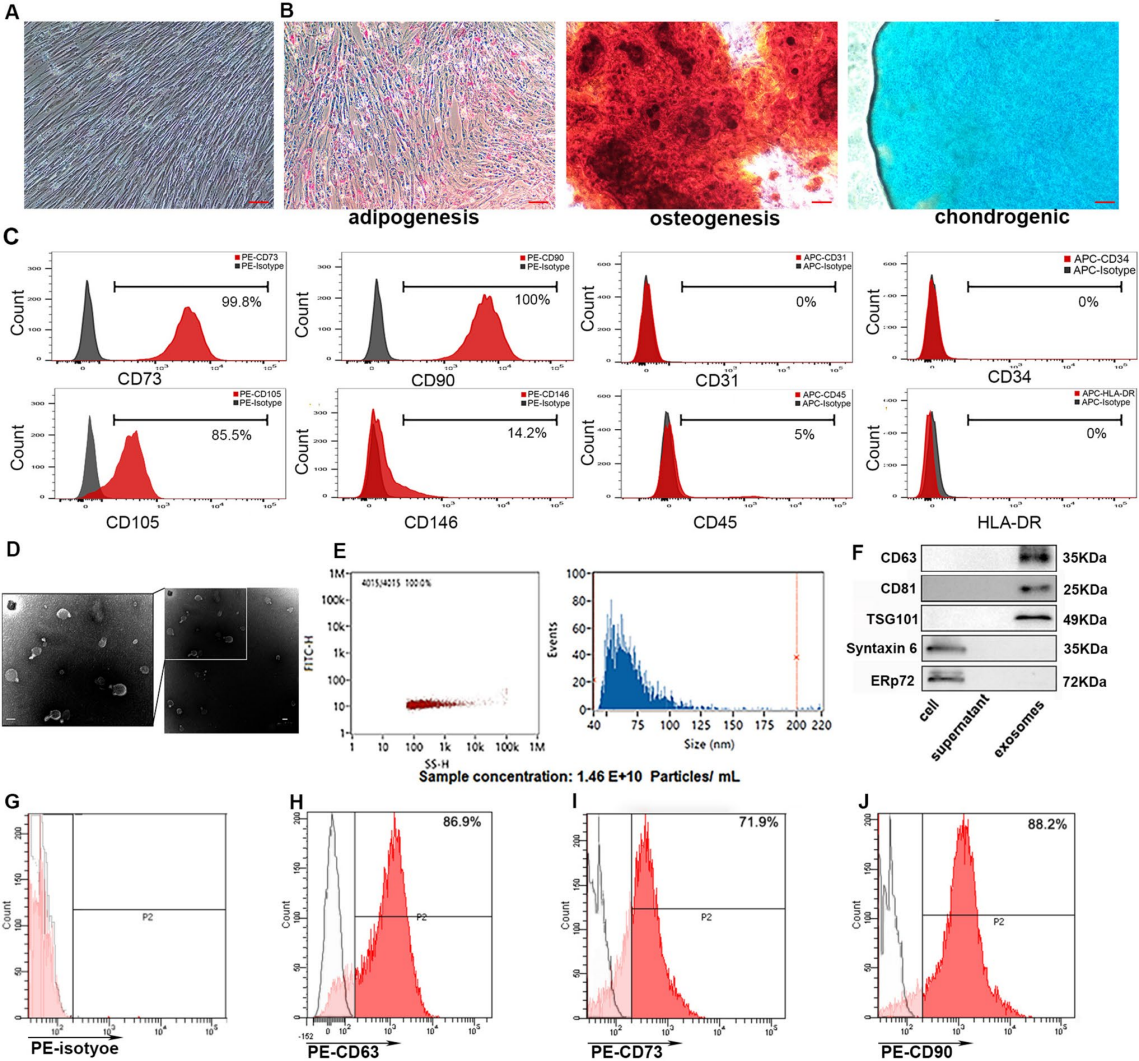
Identification of human umbilical cord-derived mesenchymal stem cells (hUC-MSCs) and characterization and identification of MSC-derived exosomes. **A** Morphology of hUC-MSCs in cell culture. **B** Oil Red O staining, Alizarin Red staining, and Alcian Blue staining of hUC-MSCs cultured in adipogenesis differentiation medium, osteogenesis differentiation medium, and chondrogenesis differentiation medium after 4 w. Scale bar, 100 μm. **C** Flow cytometry analysis of the expression of the surface markers CD73, CD90, CD105, CD146, CD31, CD34, and CD45. **D** The morphology of MSC-derived exosomes was identified under a transmission electron microscope to have a diameter of approximately 100 nm. Scale bar, 100 nm. **E** Particle concentration of extracellular vesicles from MSCs detected by nanoFCM (left panel). The size distribution of MSC-derived exosomes was measured by nanoFCM. Mean value, 69.04 nm (right panel). **F** The expression of specific surface markers of exosomes (CD63, CD81, and TSG101) and contamination markers (Syntaxin6 and Erp72) was detected by Western blot in MSC cell lysate, culture medium post-exosome isolation, and MSC-derived exosomes. Surface expression of exosome-enriched tetraspanin CD63 (**H**) and MSC surface markers CD73 (**I**) and CD90 (**J**) on exosome membranes was analyzed by FCM. The PE-isotype was used as a negative control (**G**)

We first characterized the identities of hUC-MSCs. The human umbilical cords were cultured for more than 4 weeks and spindle-shaped fibroblasts were observed (Fig. 1A). In addition, the multilineage differentiation potential of MSCs was demonstrated by Oil Red O staining, Alizarin Red staining, and Alcian Blue staining (Fig. 1B). MSCs are known to express cell surface markers like cluster of differentiation CD29, CD44, CD73, CD90, and CD105, but to lack CD14, CD34, CD45, or human leucocyte antigen-DR (HLA-DR) [32]. The flow cytometry analysis revealed that our isolated hUC-MSCs were positive for CD73, CD90, CD105, and the stemness marker CD146, but negative for CD34, CD45, or HLA-DR indicating that the cultured cells exhibited MSC-like features (Fig. 1C).

To prepare exosomes, 500-mL MSC conditioned medium was centrifuged, and 10^10^–10^11^of exosomes were purified. Isolated MSC exosomes were validated in terms of their morphology, size, and specific markers using TEM, nanoFCM analysis, and Western blotting. Through TEM, it was observed that the exosomes exhibited typical disc shapes (Fig. 1D). The exosome diameter ranged from 50 to 150 nm based on nanoFCM analysis and the mean diameter was ~ 69.04 nm (Fig. 1E). For exosomal surface markers, MSCs, supernatant, and exosomes were loaded onto SDS-PAGE gels. Determined by Western blotting, the presence of CD63, CD81, and tumor susceptibility gene 101 protein (TSG101), which are commonly enriched in exosomes, while the absence of contamination markers, Syntaxin6 for the trans-Golgi network and Erp72 for the ER lumen, were confirmed in MSC-derived exosomes (Fig. 1F), suggesting that our isolation would not damage the characteristic of exosomes. In addition, flow cytometry revealed that the proportions of CD63 + , CD73 + , and CD90 + particles were 86.9%, 71.9%, and 88.2%, respectively, indicating that the exosomes had an MSC origin (Fig. 1G–J).

### Optimization of PMO-146b-modified exosomes

**Fig. 2:**
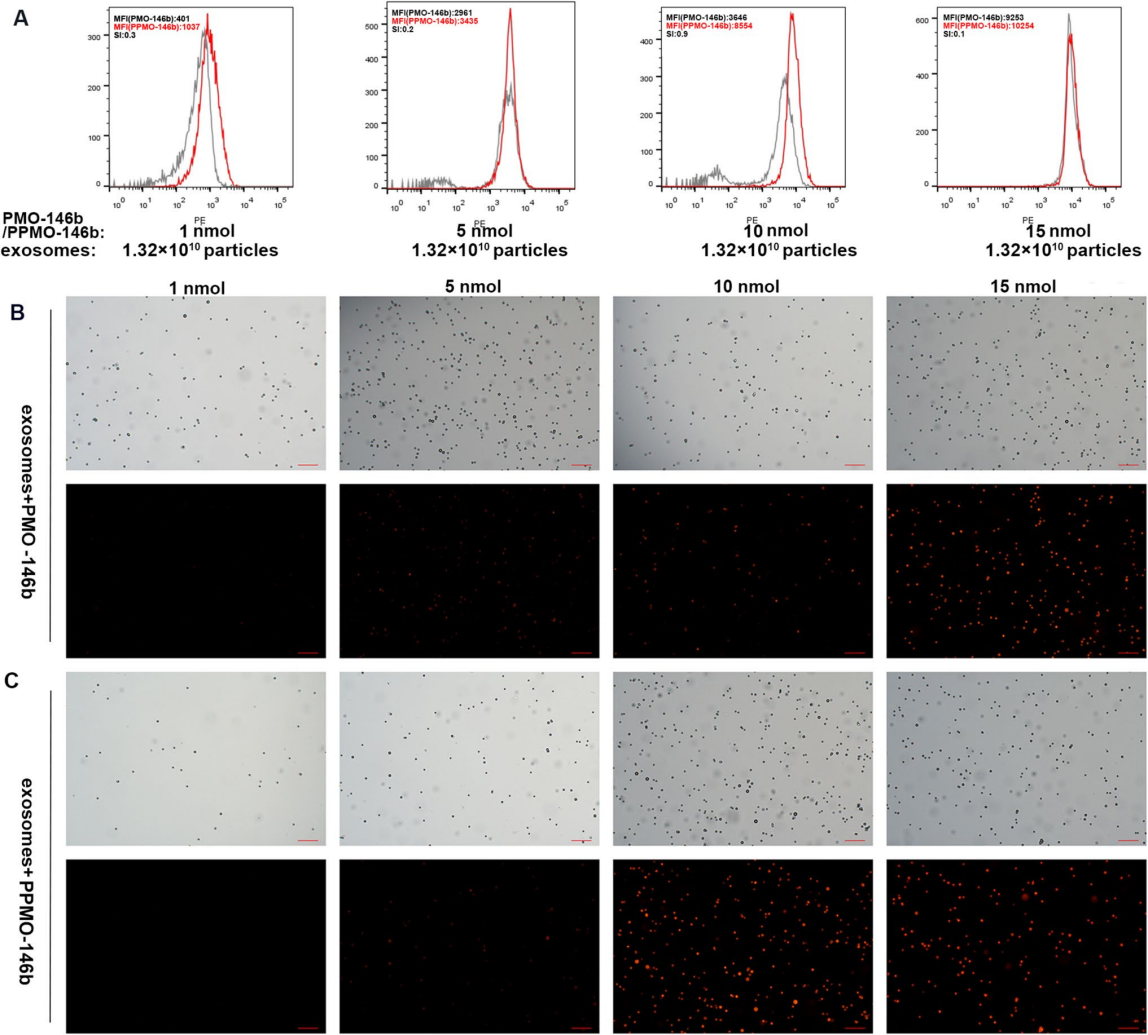
Establishment of the exosome/PMO-146b (PMO and PPMO) ratio via detection with streptavidin-PE. Aldehyde/sulfate latex beads were precoated with exosomes at a concentration of 1.32 × 10^10^ particles, followed by co-incubation with increasing moles of biotinylated PMO-146b with or without CP05 peptide (PMO/PPMO). **A** Detection was performed with pretitered streptavidin-PE. Capture was performed with biotin-labeled PMO or PPMO peptide. The ratio of 10 nmol PMO/PPMO/1.32 × 10^10^ exosomes produced the optimal SI at 0.9. The SI value of calculation is based on the difference in MFI between PMOs and PPMOs that were incubated with MSC-derived exosomes and the average spread of their distributions. Different concentrations of exosome-PMO-146b (**B**) and exosome-PPMO-146b (**C**) complexes were prepared using the same procedure above and were viewed under conventional fluorescence microscopy after the beads were recovered. Upper: bright field; under: fluorescence. Scale bar, 200 µm

In our previous study, we have determined that miR-146b promoted epithelial–mesenchymal transition in CRC indicating its potential as a therapeutic target [19]. To deliver anti-miR-146b into cells effectively and safely, exosomes were turned into viable nanocarriers for transferring the PPMO-146b conjugate. As illustrated by our Graphical Abstract, the PMO or CP05 peptide-PMO (PPMO) conjugates anchored onto the exosome surface. To determine the optimal ratio of exosomes and PPMO (PPMO-146b conjugates), conventional flow cytometry was used. We successfully stained nanosized exosomal drug delivery vehicles that were loaded with PMO (PMO-146b cargo) or PPMO and measured the MFI and SI of the oligonucleotides carried by exosomes. The optimal ratio of the exosomes/PMO was determined by titrations as illustrated in Fig. 2. Increasing moles of biotinylated PMO or PPMO were co-incubated with 1.32 × 10^10^ beads/exosomes complexed with streptavidin-PE. The fluorescence intensity is a result of (1) the positive level of PMO-biotin and (2) its density on exosomes. This result is best described by the ratio of MFI of stained PPMO to MFI of stained PMO, the significance of which is determined by the SI. MFI of exosomes treated with 10 nmol PPMO was 2.3-fold higher than that of PMO. Meanwhile, 10 nmol PMO/PPMO showed the highest SI value 0.9, reflecting a significant separation between PPMO and PMO peaks (Fig. 2A). The optimal ratio was also demonstrated using fluorescence microscopy (Fig. 2B, C). These results indicated the potential of CP05 peptide-conjugated exosomes to be appropriate delivery vehicles for various types of therapeutic oligonucleotide agents including but not limited to PMO-146b.

### ePPMO-146b inhibited the proliferation of CRC cells

**Fig. 3:**
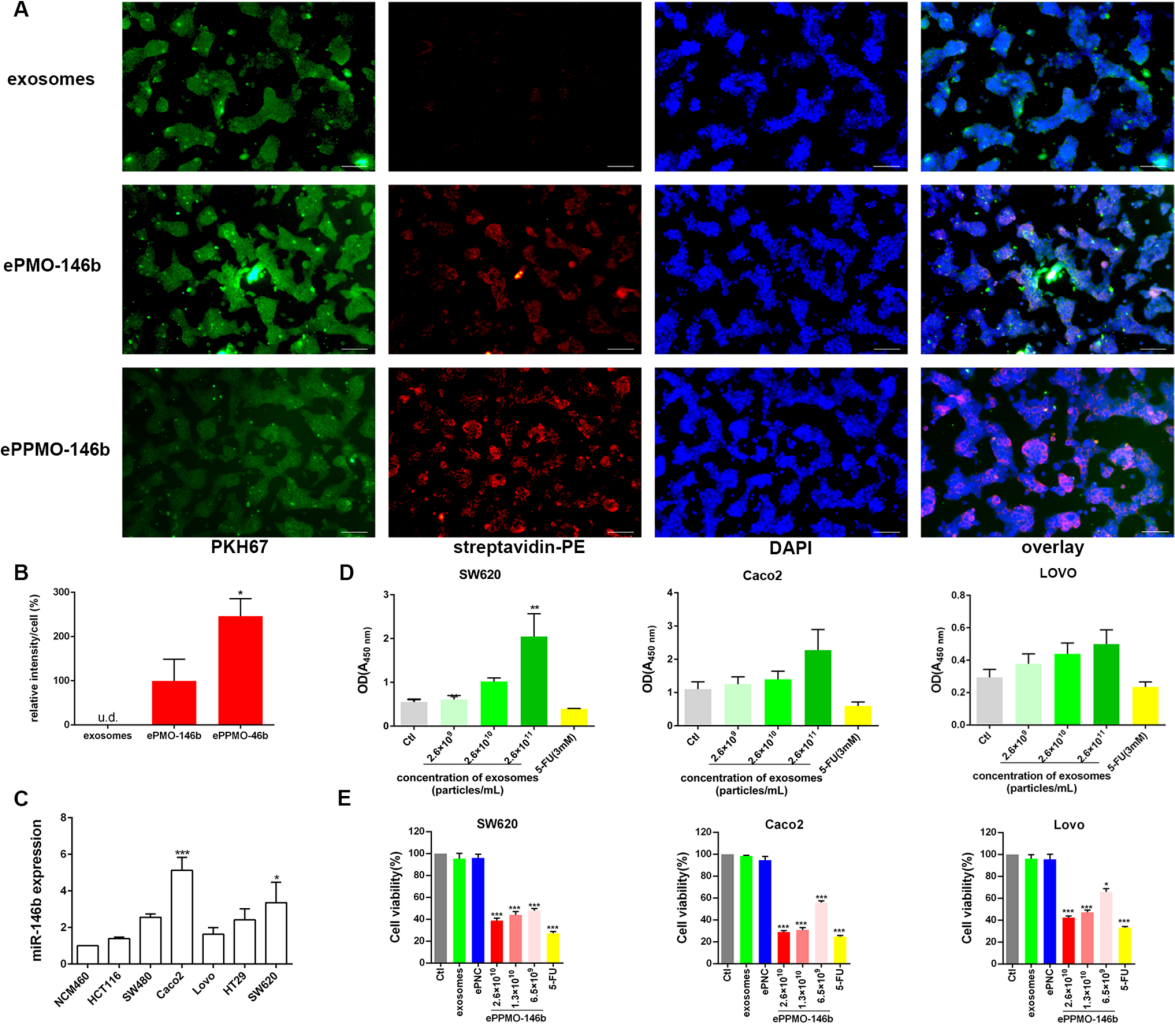
ePPMO-146b could be internalized by recipient cells and inhibited CRC cell viability. **A** MSC-derived exosomes labeled with PKH67 were co-incubated with biotin-labeled PMO-146b with or without CP05 and were subsequently added to SW620 cells, followed by incubation with streptavidin-PE and visualization under fluorescence microscopy. Note the intracellular localization of the labeled exosomes. The control involved the uptake of exosomes by SW620 cells in the absence of PMO-146b. Scale bar, 100 µm. **B** The relative intensity per cell of exosomal uptake by SW620 cells normalized to the ePMO-146b group. *P < 0.05 by one-way ANOVA. **C** qRT-PCR illustrated that the expression of miR-146b was upregulated in six CRC cell lines compared with NCM460 cells. **D** The viabilities of the SW620, Caco2, and Lovo cells exposed to native exosomes were detected by a CCK8 assay to determine the optimal concentration. Averaged data (mean ± SEM, *n* = 3) of OD at 450 nm. **P < 0.01 by one-way ANOVA. **E** The relative cell proliferation ratio was normalized to the cell index of the SW620, Caco2, and Lovo cells treated with solvent (PBS, Ctl, same as below) after 48 h. MSC-exos-loaded PMO-146b (ePPMO-146b) inhibited cell growth, as demonstrated by the CCK8 assay. Averaged data (mean ± SEM, *n* = 3) of OD at 570 nm. *P < 0.05, **P < 0.01, and ***P < 0.001 by one-way ANOVA

To investigate whether host cells could take up exosomes and whether CP05 affects the cellular internalization of exosomes, SW620 cells were incubated with PKH67-labeled (green fluorescence) native exosomes or biotinylated PMO-146b-loaded PKH67-labeled exosomes. After 12 h of cytoplasmic green staining, it was observed that a great number of exosomes were taken up by the SW620 cells (Fig. 3A, upper line). Compared with native exosomes, ePMO-146b and ePPMO-146b achieved significantly higher cellular uptakes of PMO in SW620 cells, suggesting that MSC-derived exosomes were able to efficiently deliver PMO to recipient cells (Fig. 3A, middle line and bottom line). In the SW620 cells treated with ePPMO-146b (Fig. 3A, bottom line; Fig. 3B), significantly stronger streptavidin-PE signals (red fluorescence) were dispersed with a punctate pattern in the cytoplasm compared with those of the ePMO-146b group (Fig. 3A, middle line), indicating that using CP05 to anchor cargo to exosomes could facilitate the cellular internalization of oligonucleotides.

To determine whether exosomes that are loaded with CP05-modified PMO-based anti-miR-146b oligonucleotides exert their inhibitory function in CRC, appropriate cell lines were screened, and the cellular viabilities of these lines were assessed. Zhu et al*.* and Shi et al*.* reported that an aberrant upregulation of miR-146b-5p was observed in CRC tissue of 49 patients; high miR-146b-5p expression was associated with advanced stage of CRC [20, 21]. Our qRT-PCR also verified that a higher expression level of miR-146b occurred in the six CRC cell lines than in the colonic epithelial NCM460 cells (Fig. 3C). The results showed that the two highest groups were SW620 and Caco2 cells, with fold changes of 3.36 ± 1.12 and 5.13 ± 0.71, respectively. As a result, SW620 and Caco2 cells were selected to investigate the therapeutic effect of ePPMO-146b. Native exosomes that are derived from MSCs may promote the proliferation of parental cells [33, 34]. Therefore, a dose-titration study of MSC-derived exosomes was performed using a CCK8 assay. Among the six CRC lines, Lovo cells had the strongest protumorigenic properties in xenograft nude mice in our laboratory; thus, the viability of Lovo cells was also examined. We then optimized the dose of native exosomes in vitro under various conditions and 5-FU (3 mM) was used as a positive control. MSC-derived exosomes at a concentration of ~ 10^11^ particles/mL accelerated cellular proliferation at 48 h, while up to ~ 10^10^ particles/mL MSC-derived exosomes did not have this effect (Fig. 3D).

We then investigated the role of ePPMO-146b in the proliferation of SW620, Caco2, and Lovo cells (Fig. 3E). Cellular viability was significantly decreased following a treatment with various concentrations of ePPMO-146b for 48 h in a dose-dependent manner (Fig. 3E). The robust inhibitory effect of ePPMO-146b was in a concentration of 2.6 × 10^10^ particles/mL (viability rate, 61.28% for SW620, 71.00% for Caco2 and 57.69% for Lovo) (Fig. 3E). On the other hand, ePNC had no significant effect on cellular proliferation, which was probably due to the minimal cytotoxicity of this exosomal delivery system. According to the calculations and the results above, the optimal exosome concentration for the in vitro study was 2.6 × 10^10^ particles/mL.

### ePPMO-146b inhibits CRC cell migration by hampering epithelial–mesenchymal transition

**Fig. 4:**
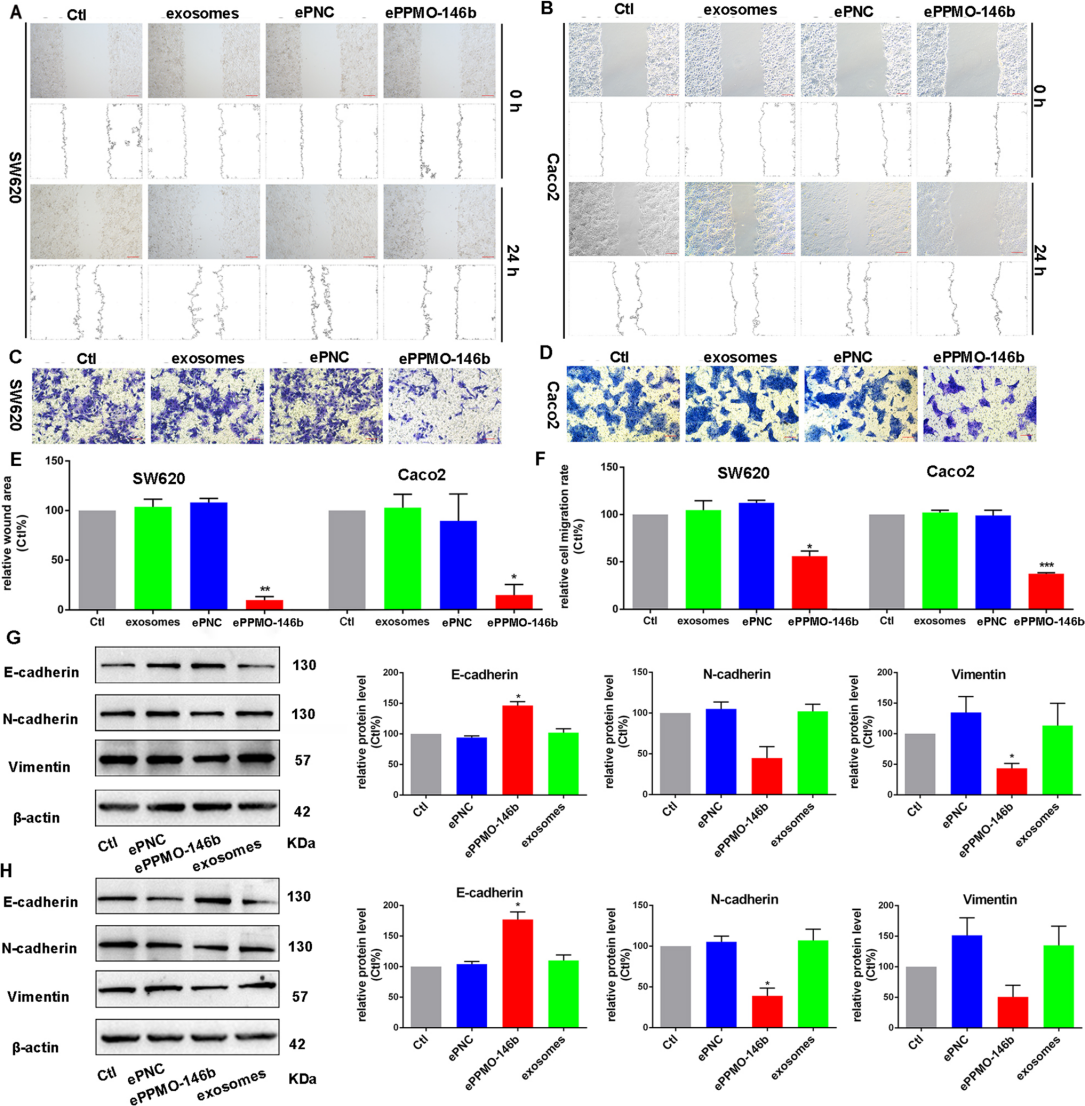
Inhibitory effect of ePPMO-146b on epithelial-mesenchymal transition in SW620 and Caco2 cells. SW620 cells (**A**) and Caco2 cells (**B**) were treated with native exosomes, ePNC, and ePPMO-146b for 24 h. ePPMO-146b significantly inhibited the migration of SW620 cells (**A**) and Caco2 cells (**B**), as demonstrated in a wound healing assay. Scale bar, 200 μm. ePPMO-146b successfully reduced the migration ability of SW620 cells (**C**) and Caco2 cells (**D**), while the native exosomes did not, as indicated by the transwell assay. Scale bar, 100 μm. **E** Column charts were plotted based on the averaged data (mean ± SEM, *n* = 3) from the wound healing assay, which shows the effective suppression effect of ePPMO-146b on migration. *P < 0.05 and **P < 0.01 by one-way ANOVA, Dunnett’s test: compared with Ctl. **F** Column charts were plotted based on the averaged data (mean ± SEM, *n* = 3) from the transwell migration assay, which shows the effective suppression effect of ePPMO-146b on migration. *P < 0.05 and ***P < 0.001 by one-way ANOVA, Dunnett’s test: compared with Ctl. The effects of ePPMO-146b on the expression of epithelial-mesenchymal transition markers in SW620 (**G**) and Caco2 cells (**H**) were observed by Western blot analysis. β-Actin was used as a sample loading control. ePPMO-146b increased E-cadherin but decreased N-cadherin and vimentin compared with Ctl. Averaged data (mean ± SEM) from three independent experiments. *P < 0.05 by one-way ANOVA, Dunnett’s test: compared with Ctl

A decrease in the migratory and invasive abilities of the CRC cells treated with AMO (anti-miRNA oligonucleotides)-miR-146b was observed in previous study [19–21], but whether ePPMO-146b exerted equal or even stronger effects remained unclear. Hence, wound healing and transwell assays were performed. As depicted in Fig. 4A, B, and E, ePPMO-146b at an exosome concentration of 2.6 × 10^10^ particles/mL reduced the migration of SW620 and Caco2 cells by 90.05% and 85.27%, respectively. And the wound healing index was gradually declined as the ePPMO-146b was stepwise withdrawn (exosome concentration at 2.6 × 10^10^, 1.3 × 10^10^, and 6.5 × 10^9^ particles/mL), indicating the dose-dependent inhibition manner of ePPMO-146b in both SW620 and Caco2 cells (Supplementary Fig. 1A, B, E, and F; *P* < 0.001). Likewise, transwell assays revealed that ePPMO-146b-treated CRC cells resulted in a significant decrease in migration of 44.01% for SW620 cells and 62.37% for Caco2 cells (Fig. 4C, D, and F), and the inhibitory potencies were gradually alleviated when intensity of ePPMO-146b treatment decreased (Supplementary Fig. 1C, G, and H; *P* < 0.05).

We previously reported that miR-146b can stimulate epithelial-mesenchymal transition promoting CRC cell invasion and metastasis [19, 20]. Therefore, whether ePPMO-146b could influence the epithelial-mesenchymal transition process of SW620 and Caco2 cells is a relevant question in the current work. When ePPMO-146b was compared with ePNC and native exosomes, it increased the epithelial cell marker E-cadherin and suppressed the mesenchymal cell markers N-cadherin and vimentin (Fig. 4G, H). These results suggested that ePPMO-146b impeded epithelial-mesenchymal transition by increasing E-cadherin and decreasing N-cadherin and vimentin.

Previous studies including ours demonstrated that expression of Smad4 is directly modulated by miR-146b [35]; we therefore hypothesized that ePPMO-146b might increase Smad4 via the miR-146b-Smad4 cascade. To test this notion, we measured the protein levels of Smad4 in various concentrations of ePPMO-146b. Western blot analysis showed that application of ePPMO-146b increasingly enhanced the protein level of Smad4 in SW620 (Supplementary Fig. 1D and I).

### ePPMO-146b accumulates in tumors and suppresses CRC tumor growth in vivo

**Fig. 5:**
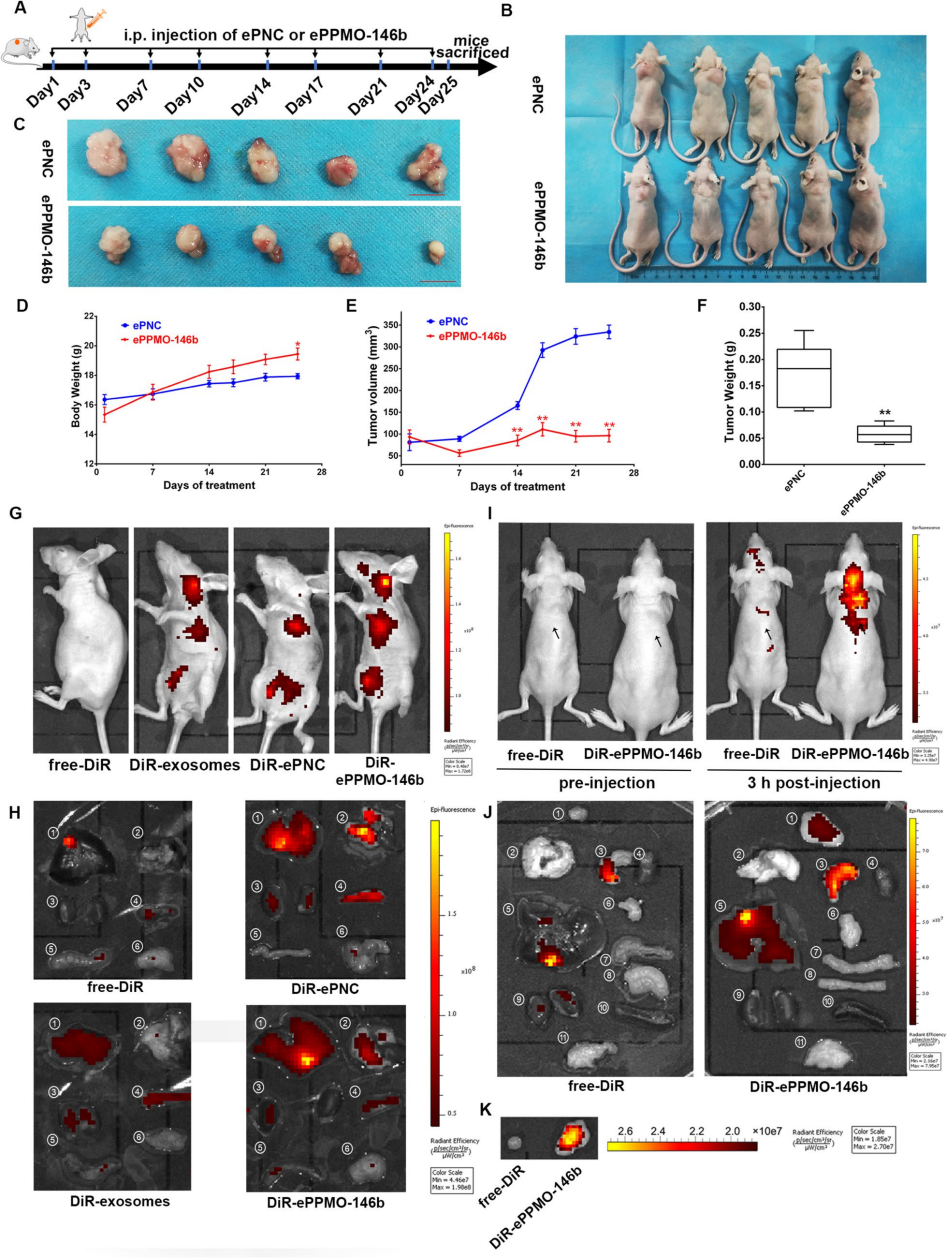
In vivo antitumor effect and the biodistribution of ePPMO-146b in mice. **A** Schematic representation of the treatment (ePNC or ePPMO-146b) in Lovo tumor-bearing nude mice via i.p. administration. **B** Representative photograph of Lovo xenograft nude mice treated with ePNC or ePPMO-146b at the end point. Exosome doses, 5.0 × 10^10^ particles/kg. **C** Photograph of the tumors dissected from xenograft mice after the last treatment. Scale bar, 1 cm. **D**–**F** Body weight and tumor growth during the follow-up are shown. The mouse weight was unaffected by PMO-146b-loaded exosomes. The tumor growth curve indicated the suppression of tumor growth by ePPMO-146b. Averaged data (mean ± SEM) from five independent experiments. *P < 0.05 and **P < 0.01 by one-way ANOVA, Dunnett’s test: compared with ePNC. **G** Representative IVIS images of the live mice injected with free-DiR-treated control, untargeted DiR-exosomes, DiR-ePNC, and DiR-ePPMO-146b via i.p. at 3 h post-injection. **H** Representative IVIS images of the different organs harvested at 6 h following the i.p. infusion of DiR-labeled ePNC or ePPMO-146b in non-tumor-bearing nude mice (1 liver, 2 heart and lung, 3 kidney, 4 spleen, 5 intestine, 6 reproductive organ). The control groups received DiR dye alone (free-DiR) to determine the background. **I** Fluorescent images of LoVo tumor-bearing mice 3 h post-injection of free DiR or DiR-ePPMO-146b. The arrow indicates where the tumor was located. **J** Fluorescent images of the major organs (6 h post-injection) harvested from mice (1 tumor, 2 lung, 3 stomach, 4 heart, 5 liver, 6 pancreas, 7 intestine, 8 colon, 9 kidney, 10 spleen, 11 reproductive organ). **K** Representative IVIS images of the tumors at 3 h post-injection of DiR-labeled ePPMO-146b

Considering that ePPMO-146b resulted in tumor suppression in vitro and was efficiently taken up by CRC cells, ePPMO-146b or ePNC at a dosage of 5.0 × 10^10^ particles/kg were intraperitoneally administrated into Lovo tumor-bearing mice twice a week for 24 days (Fig. 5A). Compared with the ePNC group, ePPMO-146b significantly impeded tumor growth (tumor weight inhibitory rate % = 64.71% and tumor volume inhibitory rate % = 71.12%; Fig. 5B–E). The antitumor activities of ePPMO-146b are summarized in Table 2. We then detected the expression of E-cadherin and vimentin expression for epithelial-mesenchymal transition using IHC in the mouse tumor samples. The result revealed that the epithelial marker E-cadherin was found to be upregulated while the mesenchymal marker N-cadherin downregulated by ePPMO-146b treatment (Supplementary Fig. 2A). Of note, this exosome-mediated delivery complex did not exert systemic toxicity to tumor-bearing mice, as evidenced by stable the body weight and no deaths observed after the ePNC or ePPMO-146b treatment (Fig. 5D). In addition, there were no obvious tissue lesions in other visceral tissues upon H&E staining by examining the liver, spleen, kidney, lung, and heart of mice, indicating the indistinct toxicity of ePPMO-146b to other organs (Supplementary Fig. 2B).

**Table 2:** Antitumor activity of ePPMO-146b against Lovo colon cancer xenografts in nude mouse models *n* = 5,$\overline{x}$ ± SEM

Drug administration			Toxicity			Anticancer activity			
Group	Schedule	Route	Average body weight (g,$\overline{x}$ ± *s*)		Death	Tumor weight (g)	IR (%)	Tumor volume (mm^3^)	IR (%)
			Start	Stop					
ePNC	BIW, 4 w	i.p	16.36 ± 0.34	17.94 ± 0.17	0/5	0.17 ± 0.03	**-**	334.31 ± 15.80	**-**
ePPMO-146b	BIW, 4 w	i.p	15.34 ± 0.50	19.44 ± 0.40*	0/5	0.06 ± 0.01	64.71%	96.40 ± 14.50**	71.12%

*BIW* twice a week: The significance of differences (vs*.* ePNC) was determined by one-way ANOVA with a *t* test.

*P < 0.05; **P < 0.01; ***P < 0.001(*n =* 5, ± SEM)

It has been demonstrated that the intraperitoneal injection of exosomes resulted in a stronger accumulation of drugs in tumors than that of intravenous injection [11]. Thus, vesicles were labeled with the lipophilic fluorescent tracer DiR. The fluorescence intensity of free-DiR, DiR-exosomes, DiR-ePNC, and DiR-ePPMO-146b was evaluated in non-tumor-bearing mice. DiR-ePNC, DiR-ePPMO-146b, and DiR-exosomes were mainly distributed in the liver in vivo by i.p. after 3 h and ex vivo at 6 h post-inoculation, which is consistent with a previous study on the biodistribution of exosomes [31] (Fig. 5G–H), indicating that the exosome-mediated delivery complex (ePNC and ePPMO-146b) resulted in a stronger accumulation in the liver than that of the unloaded exosome group (Fig. 5H).

Additionally, an ideal anticancer drug delivery system following systemic administration should be characterized by tumor accumulation; therefore, the in vivo biodistribution of ePPMO-146b was investigated in tumor-bearing mice. Similar to non-tumor-bearing mice, fluorescent signal was still detected in the liver while some were found dramatical accumulation in tumor sites, suggesting a specific retention of exosomes in tumors (Fig. 5I–K). All these data revealed that ePPMO-146b efficiently targeted PMO to the tumor sites while reserving its more robust accumulation in the liver compared to that of unloaded exosome.

## Discussion

In the present study, we developed a hUC-MSC-derived exosome-based drug delivery strategy by loading CP05-PMO-146b onto the surface of exosomes. ePPMO-146b successfully induced a therapeutic effect on CRC progression by inhibiting epithelial–mesenchymal transition in vitro and in vivo*,* which was in accordance with our previous work [19]. Of particular importance, this MSC-derived exosome system did not induce any cellular and systemic toxicity and was able to deliver the payload safely and effectively with excellent ability to target the tumors. The resulting hepatic accumulation also suggested that an inhibitory effect occurred on the liver metastasis of CRC. To our knowledge, ePPMO-146b represents the first potential practice of exosomes in the application of PMO-146b-based anticancer therapy to overcome the translational limitation of oligonucleotides in CRC.

Because anticancer chemotherapeutic drugs cause severe side effects, a new drug pipeline needs to minimize the toxicity of a drug while ensuring that it accumulates in target tissue. For example, to date, fluoropyrimidines are still the first-line conventional chemotherapy for metastatic CRC treatments. However, fluoropyrimidines indiscriminately destroy normal cells and cause toxic side effects, and a gradually developed multidrug resistance introduces clinical challenges to physicians [36]. Due to the lack of specific targets of chemotherapy drugs, targeted therapies, such as monoclonal antibodies and small molecule inhibitors, are sought out. In the manufacturing scale of biomedicines, the process of developing monoclonal antibodies is unsustainable and has emerged as an industrial bottleneck [37]. In addition, conventional small-molecule pharmaceuticals entail much larger, and often iterative, screening efforts, which are followed by extensive steps of optimizing medicinal chemistry parameters [1]. miRNA-based therapy appears to be a very ingenious and promising approach for gene therapy with CRC. Recently, some miRNA drugs have been approved to enter clinical trials on indications other than solid tumors, such as cutaneous T-cell lymphoma and Alport syndrome [38, 39]. Many data have demonstrated a definitive protumorigenic role of miR-146b in CRC, suggesting that ASO-miR-146b is feasible for developing a targeted drug [19–21]. To overcome the poor cellular uptake of oligonucleotide drugs, nanotechnology has been developed for miRNA or ASO delivery. miRNA delivery is optimized using either viral or nonviral methods. Viral delivery of synthetic miRNA has been demonstrated to be very efficient; however, immunogenicity remains a bottleneck for clinical application. In the context of nonviral methods, exosomes, as “natural” delivery vehicles that are nontoxic and are produced in an autologous manner, are capable of transferring therapeutic molecules (such as nucleic acids and recombinant proteins) into cancer cells. The following strategies have been developed to optimize the “cargo” loading of exosomes: (1) transfect therapeutic miRNAs or miRNA expression vectors into parental cells; then, miRNAs are subsequently enclosed in exosomes endogenously. For example, lenti-miR-128-3p was transfected into normal intestinal FHC cells, and exosomes were isolated and resensitized CRC to oxaliplatin [40]. (2) Exosomes were isolated from donor cells followed by the application of artificial internalization methods (such as electroporation, sonication, and surface functionalization with lipophilicity conjugation) to exogenously introduce therapeutic miRNAs into vesicles. For example, ASO-miRNA-221 was loaded into exosomes from MSCs by electroporation to treat CRC [41]. (3) The external membrane of isolated exosomes was chemically modified. Chemical modification does not involve tedious gene engineering and the low efficiency of internalizing; thus, the technique is deemed as an easily attainable alternative that is highly efficient in loading exosomes with therapeutics. Regarding the in vivo digestive circumstance, the ASO synthesized with PMO is an ideal resolution. PMOs are a charge-neutral class of antisense agents that interfere with target transcripts either by binding or by sterically blocking the assembly of translation machinery, resulting in enhanced oligonucleotide drug delivery [42]. Although PMOs are a new generation of antisense oligomers with a relatively high specificity and efficacy, the transportation of PMOs into cells can only be implemented via physical methods with low bioavailability rather than with transfection reagent s[24]. Importantly, Gao et al. reported that the CP05 peptide (CRHSQMTVTSRL) enabled PMOs to conjugate exosomes by anchoring to CD63. In this study, we addressed the most relevant challenges in applying exosomes as vehicles to load PMO-146b for directed colon cancer therapy (Figs. 4 and 5), and the process from cellular uptake (Fig. 3A, B) to biodistribution and safe systemic delivery was included (Fig. 5). Despite the remarkable advances and successes of exosomes in the field of drug delivery, there are increasing concerns about cellular production and reliable sources of EVs [8]. MSCs are the only human cell type amenable to scalable production of exosomes and have been exploited to clinical grade [11]. Since it is vital to perform repeatable measurement of EV concentration for further application in drug delivery, critical quality attributes (CQAs), such as particle size distribution (PSD), particle concentration (quantification of particle number per unit of volume), and phenotype, are indispensable [43]. Different techniques have been applied to measure physical EV properties, and these techniques include multiangle dynamic light scattering (MADLS), asymmetric flow field flow fractionation coupled with multiangle light scattering (AF4-MALS), centrifugal liquid sedimentation (CLS), nanoparticle tracking analysis (NTA), tunable resistive pulse sensing (TRPS), and high-sensitivity nanoflow cytometry (nanoFCM) [43]. NTA is the most frequently used approach, but Vogel et al. reported that NTA had to be averaged over three independent runs with CVs (CV = standard deviation/mean, 19%), while nanoFCM was performed just once [44]. Additionally, nanoFCM reached a nominal resolution below 100 nm through a combined implementation of fluorescence triggering, narrow laser beam, sensitive detectors, and instrument configuration [44]. Accordingly, in this study, nanoFCM was applied to verify the order of magnitude of exosomes (Fig. 1B).

Exosomal cargoes have been found to correlate with the physiological state of donor cells. Isolated tonsil-derived MSC-secreted EVs possessed a tumor suppressive effect on the human liver cancer cell line HepG2 [45]. Due to their multipotency, MSC-derived exosomes could also be tumorigenic [46]. Bone marrow MSC–derived exosomes favored tumor growth in vitro and in vivo [47]. Compared to other MSC origins, hUC-MSCs are preferred for cell-free therapies because they have the lowest tumorigenic ability, do not have ethical concerns, and utilize a noninvasive isolation procedure; in addition, hUC-MSCs exhibit a lower immunogenicity, faster self-renewal ability, more stable doubling time, and higher proliferation potency [48]. Nevertheless, there is no evidence of whether hUC-MSC-derived exosomes promote CRC development. Therefore, the appropriate concentration of hUC-MSC exosomes was ascertained in this study by CCK8 assays (Fig. 3E). hUC-MSCs are well suited for the mass production of exosomes at 10^10^ particles/mL, which is ideal for drug delivery in vitro.

We confirmed the tumorigenic role of miR-146b in CRC, whereas the antitumor effect of hUC-MSC-derived exosome-based delivery of PMO-146b is still unknown. Based on this issue, the CP05 peptide acted as a bridge to link PMOs and exosomes to facilitate the systemic delivery of PMO-146b. Unfortunately, we could not directly quantify the delivery efficiencies of PMO-146b loading exosomes before or after the drug delivery complex incorporating into colorectal cells by qRT-PCR because of the charge-neutral phosphoramidate linkages and blocked 3′-end of PMO [24, 49, 50]. The disclosure of PMO’s dissociation rate or release time profile deserves further study and is currently under investigation in our laboratory. On the other hand, due to the application of unmodified PMO which is greatly limited by inefficient *in vivo* delivery unless a relatively high dose was administrated, the naked PMO-146b alone is not expected as a qualified control [51]. In this study, the PKH67-labeled MSC exosomes that were co-incubated with biotin-conjugated PMO-146b and detection by streptavidin-conjugated PE via flow cytometry indirectly verified that the MSC exosomes were efficiently loaded with PMO (Fig. 2) and delivered into SW620 cells (Fig. 3A). When cellular ePPMO-146b reached the definite concentration, CRC cell viability was obviously inhibited (Fig. 3E), and consistent with anti-miR-146b, ePPMO-146b resulted in an inhibitory effect on the epithelial-mesenchymal transition phenotypes of CRC cells (Fig. 4). Simultaneously, ePPMO-146b inhibited the growth of transplanted tumors to a certain extent compared to ePNC (Fig. 5A–F). Determined in tumor tissues, expression profiles of E-cadherin and vimentin agreed with the in vitro experimental findings, indicating that ePPMO-146b possibly inhibited epithelial-mesenchymal transition in vivo (Supplementary Fig. 2A)*.*

The major advantages of the exosome delivery system include its minimal toxicity and the resulting hepatic accumulation in vivo. Regarding the exosome, previous studies revealed that no significant histological tissue damage or cytotoxicity to organs was observed in exosome-treated animals [52]. As CP05 peptide targets CD63 and CD63 well expressed in different cells, this may lead the CP05 binding with the membrane aimlessly, resulting in a weak selectivity of CP05 for cells. Actually, this concern is a reason for us choosing exosome to in vitro prepare CP05-ASO-exosome complex. We can load the exosomes with CP05-ASOs by virtue of the CD63 specific binding nature, make sure all of the CP05-ASOs have been bound to exosomes and no redundant CP05-ASOs exist in the system before treating the animals, and circumvent the universal binding of CP05 to somatic cells. Importantly, many studies have in depth investigated the in vivo safety of CP05 and PMO molecule. For example, Gao et al*.* reported that treatment of CP05-PMO-exosome complexes (EXO_PMO_) elicited undetectable toxicity to mdx mice, evidenced by no changes in blood alkaline phosphatase, urea nitrogen, and creatinine and no evidence of liver or renal toxicity. Systematic administration of CP05-PMO or CP05-engineered exosomes also induced no significant inflammatory response in vivo [25]. To date, excellent safety profile of PMOs has been achieved in preclinical studies using nonhuman primates and rodents when long-term and high doses of PMOs were delivered. No drug-related effects were noted on survival, clinical observations, body weight, food consumption, ophthalmoscopic and electrocardiographic evaluations, hematology, clinical chemistry, urinalysis, organ weights, and macroscopic evaluations in cynomolgus monkeys or mice [53, 54]. In our study, the anti-miR-146b ASO was also synthesized as PMO. The exosome-mediated delivery complex did not exert systemic toxicity to tumor-bearing mice, as evidenced by the stable body weight and no deaths observed after the ePNC (control) or ePPMO-146b treatment (Fig. 5D). We also verified that ePPMO-146b has no obvious toxicity to normal organs by H&E staining (Supplementary Fig. 2B). Taken together, these findings demonstrate that CP05-PMO conjugates enable safe delivery of therapeutic cargos, like miR-146b via exosomes.

Our preliminary experiments determined the systemic distribution of exosomes in non-tumor and tumor-bearing mice, revealing exosomes mainly located in the liver and the tumor (Fig. 5H, J). Wiklander et al*.* reported that biodistribution profile of dye-labeled exosomes remained largely unchanged during the first 24 h and significantly declined at 48 h post-administration [31]. However, dyes yielded inaccurate spatiotemporal information regarding the fate of the exosomes in vivo because of the long half-life and recyclability, resulting in intact and visible presence in organs over time [55]. Monitoring exosomal signal in organs and biofluids by a membrane reporter, Lai et al*.* revealed that exosomes first underwent a rapid distribution phase with a short half-life of < 30 min in most tissues, following a longer elimination phase within 6 h, which were much faster than using dye-labeled exosomes [55]. Due to the inconsistency and possible delayed tissue accumulation of dyes, we chose to determine exosomal biodistribution at earlier time points (3 h and 6 h post-injection, respectively). Consistent with our results, previous studies reported that following systemic administration, DiR-labeled exosomes localized predominantly in the liver and spleen with the highest fluorescence intensity, but low signal in the lungs and negligible signal in other organs [56], mainly due to the presence of abundant macrophages [57]. Of particular importance, in our study, the exosomal enrichment in the liver was enhanced when exosomes were loaded with PPMO-146b or ePNC compared to unloaded exosomes, indicating that exosome-mediated delivery complex is a precise delivery platform for ASO (Fig. 5G–J) and the therapeutic potential of the complex to inhibit hepatic metastasis of CRC because the liver is the major target organ of metastatic CRC.

## Conclusions

Our findings demonstrated that hUC-MSCs are a reliable source of exosomes and that MSC exosomes are among the best candidates for miRNA-based therapeutics. In the present study, we described a hUC-MSC-derived exosome-based delivery system that loaded anti-miR-146b, namely, ePPMO-146b, into CRC tumor tissue. ePPMO-146b significantly inhibited CRC by inhibiting epithelial–mesenchymal transition in vitro and in vivo and was a safe and effective CRC treatment. Our results provide a treatment modality for miRNA-based or even oligonucleotide-based therapeutics in cancer by applying hUC-MSC-derived exosomes as vehicles.

### Supplementary Information

Below is the link to the electronic supplementary material.

Supplementary file1 (DOCX 14 KB)

## Data Availability

The datasets generated and/or analyzed during the current study are available from the first and the corresponding author on reasonable request.

## Abbreviations

hUC-MSC: Human cord umbilical mesenchymal cell
ASO: Antisense oligonucleotide
AMOs: Anti-miRNA oligonucleotides
siRNAs: Small interfering RNAs
MSCs: Mesenchymal stem cells
HCC: Hepatocellular carcinoma
CRC: Colorectal cancer
BM-MSCs: Bone marrow MSCs
ASO: Antisense oligonucleotides
PMO: Phosphorodiamidate morpholino oligonucleotide
NanoFCM: Nanoflow cytometry
TEM: Transmission electron microscopy
HLA-DR: Human leucocyte antigen-DR
TSG101: Tumor susceptibility gene 101 protein
FDA: US food and Drug Administration
