# Supplementary material for: Anticancer effect of hUC-MSC-derived exosome-mediated delivery of PMO-miR-146b-5p in colorectal cancer
Source: Drug Deliv Transl Res. 2023 Nov 17;14(5):1352–69. doi: 10.1007/s13346-023-01469-7 (PMC10984892; doi:10.1007/s13346-023-01469-7)
Supplement: Supplementary file 1 — Supplementary file1 (DOCX 14 KB) [file 13346_2023_1469_MOESM1_ESM.docx]

**Supplementary Figure 1.** Inhibitory effects of ePPMO-146b on the migration potential of SW620 and Caco2. The wound healing assay was used to determine the concentration-dependent inhibition of metastasis in (A) SW620 and (B) Caco2 cells during treatment of ePPMO-146b. Scale bar: 200 μm. (C) Migration of ePPMO-146b-treated SW620 and Caco2 cells was evaluated by transwell assay. Scale bar: 100 μm. Three independent experiments were examined and representative images are presented. (D) Western blot results of Smad4 expression levels after adding various doses of ePPMO-146b to SW620 cells for 24 h. (E-I) Column charts were plotted based on the averaged data (mean±SEM, n=3) from the wound healing assay, the transwell migration assay and Western blot. **P* < 0.05, ***P* < 0.01, ****P* < 0.001 *vs.* Ctl.

**Supplementary Figure 2.** Histological examination of anti-tumor effect and tissue damage after several injections. (A) Immunohistochemistry staining of E-cadherin and vimentin in xenograft tissues in epNC and ePPMO-146b groups. Scale bar: 100 μm. (B) Hematoxylin and eosin (H&E) staining of liver, spleen, kidney, lung and heart tissue sections from tumor-bearing mice treated with ePNC and ePPMO-146b following 24 days administration. Scale bar: 100 μm.
